# Degradation of β-Carbolines Harman and Norharman in Edible Oils during Heating

**DOI:** 10.3390/molecules26227018

**Published:** 2021-11-20

**Authors:** Wei Liu, Zhaoyu Yang, Lili Shi, Ziyu Cui, Yun Li

**Affiliations:** 1 College of Food Science and Technology, Henan University of Technology, Lianhua Street, Zhengzhou 450001, China; 201892085@stu.haut.edu.cn (Z.Y.); shililislla@163.com (L.S.); cuiziyu155@163.com (Z.C.); 2 Key Laboratory of Agro-Products Safety & Quality of the Ministry of Agriculture, Institute of Quality Standards & Testing Technology for Agro-Products, Chinese Academy of Agricultural Sciences, No. 12, Zhongguancun South Street, Beijing 100081, China

**Keywords:** β-carbolines, harman, norharman, degradation, vegetable oil blend, heating

## Abstract

The β-carbolines, mainly including harman and norharman, are a group of naturally occurring, plant-derived alkaloids, and are also considered as nonpolar heterocyclic aromatic amines. Sesame seed oils contain a high level of β-carbolines (harman and norharman). In China, sesame seed oil blends are one of the most popular types of vegetable oils blends, which can be used as cooking oils or frying oils. Thus, it is meaningful to investigate the degradation of β-carbolines (harman and norharman) in sesame seed oil blends as frying oils during heating. In this work, the loss of harman and norharman in different types of sesame seed oil blends have been investigated. The results showed that the degradation of harman and norharman were dependent both on the type of oil blends, heating temperature and time. Harman and norharman were more degraded during heating (150 °C, 180 °C) in oleic acid-rich oil blends compared to polyunsaturated acid-rich oil blends. Mechanistic investigation suggested that the reduction in harman and norharman in oil blends during heating was mainly due to the oxidative degradation reaction between β-carbolines and lipid oxidation products. Therefore, the contents of β-carbolines (harman and norharman) in sesame seed oil blends when used as frying oils and heated can be decreased with prolonged cooking time.

## 1. Introduction

**Figure 1:**
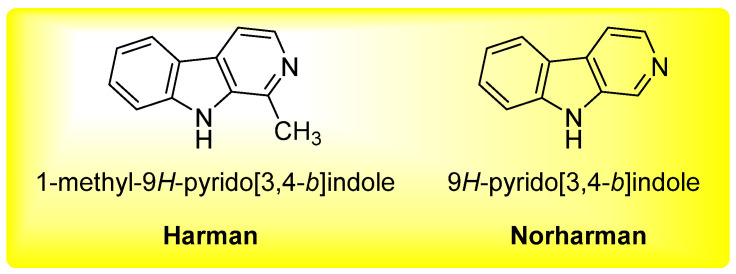
The chemical structure of β-carbolines harman and norharman.

β-carbolines, mainly including 1-methyl-9H-pyrido[3,4-b]indole (harman) and 9*H*-pyrido[3,4-b]indole (norharman) (Figure 1), are biologically active alkaloids, which are present in several plants and in thermally processed foods [1,2]. Harman and norharman, considered as nonpolar heterocyclic aromatic amines (HAAs), are produced during the treatment of proteinaceous food at heating temperatures through pyrolysis of proteins or amino acids [3,4]. Many studies have disclosed the direct correlation between enhanced cancer risk and HAAs intake [5,6]. On the other hand, some reports also showed that harman and norharman exhibited neuroactive activity in human body [1,7].

Harman and norharman can be found in many processed foods, including cookies, maize, soy and coffee [8,9]. Coffee products have high concentrations of β-carbolines (harman and norharman) [1,10]. For example, coffee grounds and instant coffee have levels of harman and norharman at 0.04–1.41 μg/g and 0.09–9.34 μg/g, respectively [8]. Some studies have shown that higher concentrations of harman and norharman in coffee were negatively correlated with Parkinson’s disease incidence [8,11]. Thus, coffee is recommended as a healthy drink in western countries. Very recently, it was found that β-carbolines (harman and norharman) were detected in some kinds of vegetable oils, such as sesame seed oils and peanut oils [12,13]. Bulk edible vegetable oils, including soybean oil, palm oil, rapeseed oil, and sunflower oil, are produced through chemical refining or pressing process [14]. Thus, a very low level of β-carbolines (harman and norharman) and other HAAs were detected in these kind of edible oils [12,13]. In China, flavored vegetable oils, including sesame seed oil and peanut oil, are very popular due to their special flavors [15,16]. For instance, sesame seeds are roasted at 180–220 °C for 30–40 min before pressing, so some special flavor compounds will be produced [17]. Thus, sesame seeds will generate β-carboline compounds after roasting at heating temperatures (>150 °C).

Vegetable oil blends are produced by blending two or more kinds of vegetable oil in a certain proportion, which can meet the requirements of nutrition, oxidative stability or flavor. Due to the flavor of sesame seed oils, vegetable oil blends containing sesame seed oil (sesame seed oil blends) is one of the most popular oil blends in China [18,19]. Though sesame seed oils have been consumed mainly due to their flavor, most pure sesame seed oils are not used directly as cooking oil (e.g., frying oil). In most cases, common vegetable oils (e.g., soybean oil, sunflower seed oil) blended with flavor oils (especially sesame seed oil) are used mainly as cooking oils (e.g., frying oils). Therefore, this study focuses on: (1) the effect of oil blend types on the degradation of β-carbolines (harman and norharman) in oil blends during heating; (2) the effect of heating temperature and time on the loss of β-carbolines (harman and norharman) in sesame seed oil blends. Moreover, the possible degradation mechanism of β-carbolines in edible oil media are discussed in this work. We believe that this work will give more insights into the degradation of β-carbolines (harman and norharman) in edible oil at cooking temperatures.

## 2. Results and Discussion

**Figure 2:**
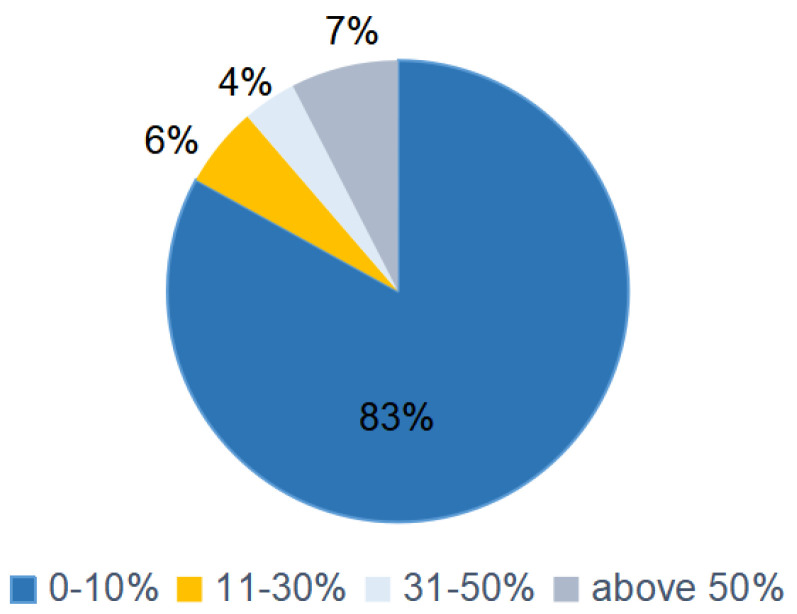
The content level of sesame oils in oil blends (53 samples).

In China, there are many kinds of commercial vegetable oil blends containing sesame seed oil (sesame seed oil blends), which can be used as cooking oil to meet the requirements of nutrition and flavor. Furthermore, the contents of sesame seed oil in different oil blends range from 1–10% (Figure 2). Therefore, four types of common bulk vegetable oils, including soybean oil (SBO), sunflower seed oil (SFO), high-oleic-acid sunflower seed oil (HOSFO) and high-oleic-acid peanut oil (HOPNO), were selected to be blended with sesame seed oil (SSO), affording four kinds of sesame seed oil blends (oil blends containing 10% sesame seed oil). Additionally, these four oil blends were used as oil media for this work (the fatty acid composition of the vegetable oils are summarized in Table S1 in Supplementary Materials).

Notably, no heterocyclic aromatic amines (including harman and norharman) were detected in the used vegetable oil samples, including SBO, SFO, HOSFO and HOPNO (Table S2 in Supplementary Materials). Indeed, refined edible oils (e.g., SBO) and pressed edible oils (e.g., SFO, HOSFO) contain no heterocyclic aromatic amines [13]. The contents of harman and norharman in the fragrant sesame seed oil (used for preparing the oil blends) were 789 μg/kg and 890 μg/kg, respectively. Therefore, the sesame seed oil blends contained the same content level of harman and norharman, which was suitable to investigate the effect of oil type on the degradation of β-carbolines (harman and norharman) in oil media during heating. Considering the common oil frying temperatures, oil blend heating was conducted at 150 °C and 180 °C, respectively.

**Figure 3:**
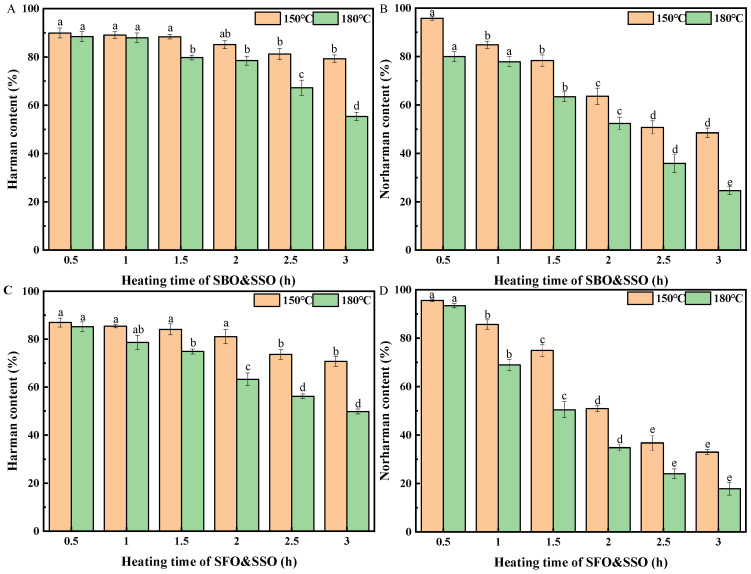
The content of harman in soybean oil blend (SBO&SSO) (**A**), the content of norharman in soybean oil blend (SBO&SSO) (**B**), the content of harman in sunflower seed oil blend (SFO&SSO) (**C**), the content of norharman in sunflower seed oil blend (SFO&SSO) (**D**). Different superscript letters indicate a significant difference at *p*-value < 0.05.

**Figure 4:**
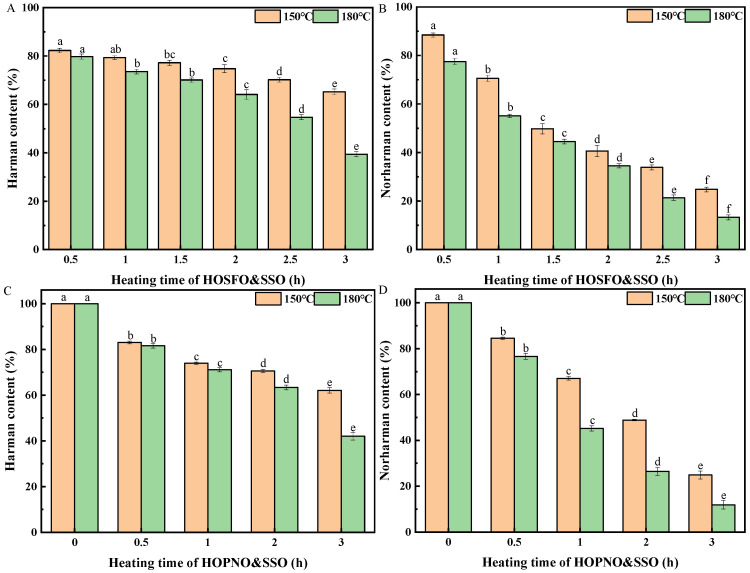
The content of harman in high-oleic-acid sunflower seed oil blend (HOSFO&SSO) (**A**), the content of norharman in high-oleic-acid sunflower seed oil blend (HOSFO&SSO) (**B**), the content of harman in high-oleic-acid peanut oil blend (HOPNO&SSO) (**C**), the content of norharman in high-oleic-acid peanut oil blend (HOPNO&SSO) (**D**). Different superscript letters indicate a significant difference at *p*-value < 0.05.

The four sesame seed oil blends (SBO&SSO, SFO&SSO, HOSFO&SSO, HOPNO&SSO) containing β-carbolines (78.88 ± 0.60 μg/kg harman and 88.90 ± 0.49 μg/kg norharman) were heated at two cooking temperatures (150 °C and 180 °C) (Figure 3 and Figure 4). Harman and norharman in the soybean oil blend (SBO&SSO) were decreased to 79% and 48%, respectively, at 150 °C with heating time prolonged to 3 h. When heating temperature was increased to 180 °C, harman and norharman in the oil blend (SBO&SSO) were decreased to 55% and 25%, respectively, with heating time prolonged to 3 h (Figure 3A,B). At 150 °C, the losses of harman and norharman in SBO&SSO were greater than 20% and 50%, respectively, after heating for 3 h. At 180 °C, the loss of harman and norharman in SBO&SSO were greater than 40% and 70%, respectively, after heating for 3 h.

The contents of harman and norharman in the sunflower seed oil blend (SFO&SSO) were decreased to 71% and 33%, respectively, when heating at 150 °C for 3 h (Figure 3C,D). When the oil blend was heated to 180 °C for 3 h, harman and norharman in the oil blend (SFO&SSO) were decreased to 50% and 18%, respectively. At 150 °C, the loss of harman and norharman in SFO&SSO were greater than 20% and 60%, respectively, after heating for 3 h. At 180 °C, the loss of harman and norharman in SFO&SSO were greater than 50% and 80%, respectively, after heating for 3 h.

The contents of harman and norharman in the high-oleic-acid sunflower seed oil blend (HOSFO&SSO) were decreased to 65% and 25%, respectively, when heating at 150 °C for 3 h (Figure 4A,B). When the oil blend was heated at 180 °C for 3 h, harman and norharman in the oil blend (HOSFO&SSO) were decreased dramatically to 39% and 13%, respectively. The loss of harman and norharman in HOSFO&SSO were greater than 30% and 70% after heating at 150 °C for 3 h, respectively. Additionally, the loss of harman and norharman in HOSFO&SSO were greater than 60% and 80%, respectively, after heating at 180 °C for 3 h.

Moreover, harman and norharman in the high-oleic-acid peanut oil blend (HOPNO&SSO) were decreased to 62% and 25%, respectively, when heating at 150 °C for 3 h (Figure 4C,D). Harman and norharman in the oil blend were decreased dramatically to 42% and 18%, respectively, when heating at 180 °C for 3 h. The losses of harman and norharman in HOPNO&SSO were greater than 30% and 70%, respectively, after heating at 150 °C for 3 h. Additionally, the losses of harman and norharman were greater than 50% and 80%, respectively, after heating at 180 °C for 3 h.

It was found that the level of decrease in harman in different oil blends at 150 °C occurred in the following order: HOSFO&SSO ≈ HOPNO&SSO > SFO&SSO > SBO&SSO. Additionally, the decrease in level of norharman in different oil blends at 150 °C followed the same order: HOSFO&SSO ≈ HOPNO&SSO > SFO&SSO > SBO&SSO. At 180 °C, the decrease in level of harman in the oil blends followed the following order: HOSFO&SSO ≈ HOPNO&SSO > SFO&SSO > SBO&SSO. At 180 °C, the decrease in level of norharman in the oil blends followed the following order: HOSFO&SSO > HOPNO&SSO ≈ SFO&SSO > SBO&SSO. Based on the above results, it was concluded that both harman and norharman were more strongly decomposed in monounsaturated fatty acid-type oil blends (e.g., HOSFO&SSO) than in polyunsaturated fatty acid-type oil blends (e.g., SBO&SSO) during heating under aerobic conditions.

The fatty acid composition of the four oil blends were summarized in Table 1. SBO&SSO contained C18:1 28.3%, C18:2 51.0% and C18:3 4.4%. SFO&SSO contained C18:1 29.0%, C18:2 58.9% and C18:3 0.2%. HOSFO&SSO contained C18:1 80.0%, C18:2 10.2% and C18:3 0.4%. HOPNO&SSO contained C18:1 72.2%, C20:1 2.0%, C18:2 10.3% and C18:3 1.1%. Although the IV (iodine value) of SBO&SSO and SFO&SSO were similar, more linolenic acid (C18:3) in SBO&SSO led to auto-oxidation more rapidly. Though the IV of HOSFO&SSO and HOPNO&SSO were similar, HOPNO&SSO contained more linolenic acid (C18:3).

A previous report has studied the stability of HAAs in pure water during heating [20]. Results indicated that the degradation losses of harman and norharman in pure water were 20% and 5%, respectively, after heating at 200 °C for 3 h. Further, another report has studied the stability of HAAs in oil during heating [21]. Results indicated that harman and norharman were much more stable than other HAAs (e.g., PhIP) in closed test tubes during heating (100–225 °C). Based on our results, the degradation losses of harman and norharman in edible oil media (under aerobic conditions) were faster than that in the closed test tubes [21], which suggested that the oxidation of edible oil indeed affected the degradation of harman and norharman. High-oleic-acid vegetable oils, including high-oleic-acid sunflower seed oil (HOSFO) and high-oleic-acid peanut oil (HOPNO), are very suitable for the food-frying industry due to their high oxidation stability. It was found that the content of harman and norharman in the oil blends rich in oleic acid (e.g., HOSFO&SSO) are decreased more rapidly than that in oil blends rich in more polyunsaturated fatty acids (e.g., SBO&SSO). In addition, the decreasing rates of norharman were slightly faster than that of harman in all tested oil blends during heating both at 150 °C and 180 °C.

**Figure 5:**
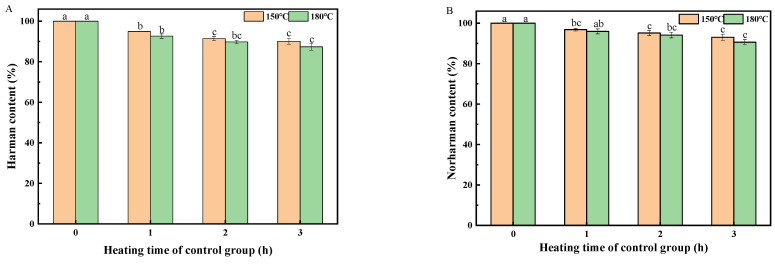
The content of harman in high-oleic-acid sunflower seed oil blend (HOSFO&SSO) under control conditions (heating without oxygen) (**A**), the content of norharman in HOSFO&SSO under control conditions (heating without oxygen) (**B**). Different superscript letters indicate a significant difference at *p*-value < 0.05.

In general, harman and norharman showed more stability than other HAAs (e.g., IQX, MeIQX, PhIP) [21]. Therefore, the possible degradation pathways for harman and norharman at heating temperature were thermodegradation and oxidative degradation (oxidation). To identify the thermostability of harman and norharman without oxygen, control experiments using HOSFO&SSO as the frying oil at 150 °C and 180 °C were conducted in the closed test tubes filled with nitrogen (Figure 5). In fact, the loss of harman and norharman in HOSFO&SSO was more significant than that in other oil blends (SBO&SSO); for example, 39% and 13% of the initial contents of harman and norharman were detected after heating at 180 °C for 3 h under air atmosphere (Figure 3 and Figure 4). In contrast, both harman and norharman exhibited good stability in heating oil (HOSFO&SSO) at both 150 °C and 180 °C (Figure 5). After heating at 150 °C for 3 h, 90% and 93% of the initial contents of harman and norharman were detected, respectively; after heating at 180 °C for 3 h, 87% and 91% of the initial contents of harman and norharman were detected, respectively. Compared with the results in Figure 4, it was found that harman and norharman were stable in closed test tubes without oxygen. Therefore, it was speculated that the loss of harman and norharman in edible oils (e.g., HOSFO&SSO) during heating was mainly attributed to the oxidative degradation of harman (norharman).

Then, the changes in acid value (AV), peroxide value (POV), anisidine value (p-AnV) and total oxidation value (TV) of the four oil blends during heating at 150 °C and 180 °C under aerobic conditions are summarized in Table 2, Table 3, Table 4 and Table 5.

The changes in AV in the four oil blends (SBO&SSO, SFO&SSO, HOSFO&SSO, HOPNO&SSO) indeed increased slightly with increasing heating time at 150 °C or 180 °C, and the final AV in the four different oil blends after heating at 180 °C were slightly higher than that at 150 °C. Peroxide value (POV) and anisidine value (p-AnV) were important indicators to measure the initial hydroperoxide concentration and secondary oxidation products concentration, respectively. During heating at higher temperature, the unstable lipid hydroperoxide underlie decomposition [21]. Thus, the changes of POV were not a suitable indicator to evaluate the oxidation process of different oil blends at higher heating temperatures in this work. However, the changes of anisidine value (p-AnV) could be used to measure the oxidation stability of different oil blends (SBO&SSO, SFO&SSO, HOSFO&SSO, HOPNO&SSO) during heating. After heating at 150 °C for 3 h, the changes of p-AnV in different oil blends followed the order: SBO&SSO > SFO&SSO > HOSFO&SSO ≈ HOPNO&SSO. After heating at 180 °C for 3 h, the changes of p-AnV in different oil blends followed the same order: SBO&SSO > SFO&SSO > HOSFO&SSO ≈ HOPNO&SSO. Indeed, higher heating temperatures led to increasing level of oxidation products (p-AnV) for all oil blends. In general, the changes of p-AnV in different oil blends were not only affected by the heating temperatures but also dependent on the type of edible oils (e.g., fatty acid composition).

Finally, the degradation mechanism of β-carbolines (harman and norharman) during heating under aerobic conditions was discussed. The thermal decomposition pathway of harman and norharman during heating in edible oil media (under aerobic conditions) could be ruled out based on the control experiments (Figure 5). In fact, harman and norharman were more stable than other HAAs at higher heating temperatures due to their higher active energies [20]. Therefore, it was concluded that the degradation of harman and norharman in edible oils were mainly attributed to the oxidative degradation of harman and norharman. There were possibly three kinds of oxidative degradation reactions for harman and norharman during heating in edible oils; the first was oxidation of harman and norharman with lipid hydroperoxides, the second was the reaction between harman (norharman) and lipid secondary oxidation products, and the third was direct oxidation of harman (norharman) with oxygen (from air). In fact, the changes of p-AnV in different oil blends followed the same order: SBO&SSO > SFO&SSO > HOSFO&SSO ≈ HOPNO&SSO (Table 2, Table 3, Table 4 and Table 5), implying that the oil blends rich in polyunsaturated fatty acids (e.g., SBO&SSO, SFO&SSO) consumed more oxygen than oil blends rich in oleic acid (e.g., HOSFO&SSO), generating more hydroperoxides (larger p-AnV value). A previous report has disclosed similar phenomenon that the effect of hydroperoxides on the stability of harman and norharman were less concentration dependent [21]. Thus, maybe the proposed three oxidative degradation pathways all play roles in the degradation of harman and norharman during heating in edible oils under aerobic conditions in this work.

## 3. Materials and Methods

### 3.1. Materials

Soybean oil, sunflower seed oil, high-oleic-acid sunflower seed oil, high-oleic-acid peanut oil and sesame seed oil were purchased from a local supermarket in China. Acetonitrile (HPLC-grade) was purchased from Thermo Fisher Scientific (Shanghai, China). Ammonium hydroxide was obtained from Kemiou Chemical Reagent Co. Ltd. (Tianjin, China). Hydrochloric acid (HPLC grade) was obtained from Kemiou Chemical Reagent Co. Ltd. (Tianjin, China). Methyl alcohol, acetic acid and *n*-hexane were of HPLC grade, and other chemicals were of analytical reagent grade. Oasis MCX solid-phase extraction cartridges (150 mg, 6 mL) were purchased from Waters (Milford, CT, USA). The water used was Wahaha purified water purchased from a local supermarket. The standards AαC (2-amino-9*H*-pyrido[2,3-b]indole), MeAαC (2-amino-3-methyl-9*H*-pyrido[2,3-b]indole), Trp-P-1 (3-amino-1,4-dimethyl-5*H*-pyrido[4,3-b]indole), Trp-P-2 (3-amino-1-methyl-5*H*-pyrido[4,3-b]indole), DMIP (2-amino-1,6-dimethylimidazo[4,5-b]pyridine), Glu-P-2 (2-aminodipyrido [1,2-a:3′,2′-d]imidazole), MeIQ (2-amino-3,4-dimethyl-imidazo[4,5-f]quinoline), MeIQx (2-amino-3,8-dimthylimidazo[4,5-f]quinoxaline), IQ (2-amino-3-methyl-imidazo[4,5-f]quinoline), PhIP (2-amino-1-methyl-6-phenylimidazo[4,5-b]pyridine), 4,8-DiMeIQx (2-amino-3,4,8-trimethyl-imidazo[4,5-f]quinoxaline), 7,8-DiMeIQx (2-amino-3,7,8-trimethyl-imidazo[4,5-f]quinoxaline) were purchased from Toronto Research Chemicals (Toronto, ON, Canada). Harman (1-methyl-9*H*-pyrido[3,4-b]indole), norharman (9*H*-pyrido[3,4-b]indole) and 4,7,8-TriMeIQx (internal standard) were purchased from Alta scientific (Tianjin, China).

### 3.2. Methods

#### 3.2.1. Preparation of Sesame Seed Oil Blends

One type of vegetable oil (360 g), such as soybean oil (SBO), sunflower seed oil (SFO), high-oleic-acid sunflower seed oil (HOSFO) and high-oleic-acid peanut oil (HOPNO), was blended with sesame seed oil (SSO, 40 g) to afford four kinds of sesame seed oil blends (SBO&SSO, SFO&SSO, HOSFO&SSO, HOPNO&SSO). Based on this method, the contents of β-carbolines (harman and norharman) in different blended oils remain at the same level, which is suitable for our investigation.

#### 3.2.2. Heating of Oil Blends

Considering the common practical oil frying temperatures (150–180 °C), the four kinds of sesame seed oil blends (SBO&SSO, SFO&SSO, HOSFO&SSO, HOPNO&SSO) were heated at 150 °C and 180 °C, respectively, for 0.5 h, 1 h, 1.5h, 2 h, 2.5 h and 3 h in an open glass container (150 × 90 mm I.D., 5.0 mm wall thickness). In the control group, 20 g blended oil (90% high-oleic-acid sunflower seed oil blended with 10% sesame seed oil) was heated for 1 h, 2 h and 3 h under the protection of nitrogen at 150 °C and 180 °C in a pressure reaction tube, respectively.

#### 3.2.3. Fatty Acid Composition of Edible Oil

The fatty acid composition was determined by conversion of oil to fatty acid methyl esters, prepared following the standard IUPAC method 2.301. Fatty acid compositions of oil blends were analyzed by Agilent Technologies 6890 N gas chromatography (GC) equipped with a 30.0 m × 250 µm × 0.25 µm BPX-70 capillary column and detected using a flame ionization detector (FID). Then, 1 µL of sample was injected under the following conditions: the nitrogen flow rate was 1.0 mL/min, the oven was programmed from the set temperature of 170 to 210 °C at 2 °C/min, the split ratio was 50:1, the GC injection temperature was 250 °C, and the detector temperature was 300 °C.

#### 3.2.4. Extraction, Purification and Analysis of HAA in Oils

Oil sample (1 g) was added with 10 μL 5mg/L 4,7,8-TriMeIQx (internal standard) and combined with 10 mL acetonitrile (containing 1% acetic acid) in a 50 mL centrifuge tube, after which the mixture was shaken for 5 min, followed by ultrasonic extraction for 10 min, and shaken vigorously for 1 min before being centrifuged at 10,000 rpm (−4 °C) for 10 min. The supernatant liquid was collected into a 50 mL centrifuge tube. The above extraction operations with acetonitrile were repeated twice. All extracts were collected together.

A solid phase extraction column cartridge—Oasis MCX (150 mg/6 mL)—was flushed in advance with 10 mL of methanol and 10 mL 0.1mol/L HCl-methanol mixed solution (80:20, *v*/*v*). All extracts were transferred to the MCX column for enrichment and purification. Then, it was washed with 10 mL water, 10 mL methanol, and 10 mL methanol/ammonia/water (25:5:75, *v*/*v*/*v*) mixed solution. Finally, 10 mL methanol/ammonia (95:5, *v*/*v*) solution was used for elution. The eluent was collected and evaporated to dryness under nitrogen. The residue was dissolved in 10 mL 5% formic acid/acetonitrile (95:5, *v*/*v*) mixture and filtered through a 0.45 μm microporous filter for LC/MS analysis.

Quantification of HAA was performed using a LC/MS according to a previous method [22]. A Shimadzu LC-20ADXR system coupled with a Triple Quad 3500 mass detector (AB SCIEX, Redwood City, CA, USA) was used to analyze the HAAs of the sample extract. Chromatographic separation was performed on an Agilent ZORBAX Eclipse XDB-C18 column (3.5 μm particle size, 150 mm × 2.1 mm i.d) maintained at 35 °C. The gradient elution was achieved with a binary mobile phase of 5% formic acid/5mM ammonium formate aqueous solution (A) and 5% formic acid/5 mM ammonium formate methanol solution (B) at a flow rate of 0.4 mL/min. The gradient elution program was as follows: 0–0.01 min, 5%B; 0.01–1.00 min, 5%B; 1.00–1.10 min, 5–60%B; 1.10–5.00 min, 60–80%B; 5.00–6.00 min, 80–95%B; 6.00–8.00 min, 95%B; 8.00–8.10 min, 95–5%B; 8.10–8.20 min, 5%; 8.20–10.00 min adjust mobile phase balance to initial state. The single injection volume was set at 5 μL.

MS analysis was carried out with a positive electrospray ionization (ESI+). Multiple reaction monitoring (MRM) conditions were automatically optimized. The capillary voltage was 5.5 kV and the ion source temperature was 550 °C. The MRM parameters for 14 HAAs and internal standard are summarized in Table S3 (see Supplementary Materials).

#### 3.2.5. Statistical Analysis

All experiments were carried out in triplicate, the mean and standard deviation (SD) for each of the determination were calculated and reported. Figure preparation was performed using Origin Pro software (Origin Lab Co., Northampton, MA, USA). The difference between groups were tested by ANOVA and Duncan’s multiple range tests. Means were compared and they were considered significant when *p* < 0.05.

## 4. Conclusions

In this work, the loss of β-carbolines (harman and norharman) in different types of edible oils (sesame seed oil blends) has been investigated. The results showed that the degradation of harman and norharman were dependent both on the type of oil blends and heating temperature and time. Harman and norharman were more degraded during heating (150 °C, 180 °C) in the oil blends rich in oleic acid compared to the oil blends rich in polyunsaturated acids. Mechanism investigation suggested that the reduction in harman and norharman in oil blends during heating was mainly due to the oxidative degradation of β-carbolines (harman and norharman) under aerobic conditions. Thus, the contents of harman and norharman in vegetable oils as frying oils during heating can be decreased rapidly with prolonged cooking time. Furthermore, oil frying provides a simple food processing method for the reduction of heterocyclic aromatic amine (e.g., harman and norharman) risk in our diet.

## Figures and Tables

**Table 1:** Fatty acid compositions (%) of different sesame seed oil blends.

	SBO&SSO	SFO&SSO	HOSFO&SSO	HOPNO&SSO
C16:0	10.72	6.86	4.31	6.23
C18:0	4.46	3.67	2.89	2.89
C18:1	28.31	29.01	80.02	72.24
C18:2	50.98	58.91	10.19	10.33
C18:3	4.36	0.21	0.37	1.13
C20:1	0	0	0	2.02
C22:0	0	0	0	2.96
C24:0	0	0	0	2.01
ΣSFA	15.18	10.52	7.20	14.09
ΣMUFA	28.31	29.01	80.02	77.21
ΣPUFA	55.33	59.12	10.56	13.48

Note: SFA, MUFA and PUFA are the abbreviation of saturated fatty acid, monounsaturated fatty acid and polyunsaturated fatty acid, respectively.

**Table 2:** Changes in AV, POV, p-AnV and TV of SBO&SSO oil blend during heating.

		0 h	0.5 h	1 h	1.5 h	2 h	3 h
150 °C	AV (mg/g)	0.68 ± 0.00 ^d^	0.71 ± 0.01 ^c^	0.78 ± 0.00 ^b^	0.79 ± 0.00 ^b^	0.79 ± 0.00 ^b^	0.96 ± 0.01 ^a^
	POV (mmol/kg)	1.40 ± 0.09 ^e^	3.87 ± 0.00 ^d^	12.93 ± 0.28 ^c^	17.95 ± 0.13 ^b^	19.35 ± 0.35 ^a^	19.66 ± 0.27 ^a^
	p-AnV	3.06 ± 0.26 ^f^	6.14 ± 0.14 ^e^	20.27 ± 0.39 ^d^	46.38 ± 0.44 ^c^	78.52 ± 0.56 ^b^	146.38 ± 0.19 ^a^
	TV	8.65 ± 0.10 ^f^	21.60 ± 0.13 ^e^	71.99 ± 1.50 ^d^	118.19 ± 0.07 ^c^	155.90 ± 0.83 ^b^	225.00 ± 1.29 ^a^
180 °C	AV (mg/g)	0.68 ± 0.00 ^d^	0.78 ± 0.01 ^c^	0.78 ± 0.00 ^c^	0.82 ± 0.00 ^b^	0.82 ± 0.01 ^b^	1.02 ± 0.00 ^a^
	POV (mmol/kg)	1.40 ± 0.09 ^d^	2.60 ± 0.09 ^c^	7.25 ± 0.65 ^a^	6.91 ± 0.18 ^a^	7.18 ± 0.01 ^a^	4.47 ± 0.09 ^b^
	p-AnV	3.06 ± 0.26 ^f^	18.07 ± 0.08 ^e^	59.31 ± 0.53 ^d^	104.24 ± 0.82 ^c^	139.38 ± 0.59 ^b^	204.17 ± 0.67 ^a^
	TV	8.65 ± 0.10 ^f^	28.47 ± 0.29 ^e^	88.30 ± 3.13 ^d^	131.90 ± 0.12 ^c^	168.11 ± 0.55 ^b^	222.04 ± 1.01 ^a^

AV, POV, p-AnV and TV are the abbreviations of acid value, peroxide value, anisidine value, total oxidation value, respectively. Different superscript letters indicate a significant difference at *p*-value < 0.05.

**Table 3:** Changes in AV, POV, p-AnV and TV of SFO&SSO oil blend during heating.

		0 h	0.5 h	1 h	1.5 h	2 h	3 h
150 °C	AV (mg/g)	0.82 ± 0.01 ^d^	0.82 ± 0.00 ^d^	0.82 ± 0.01 ^d^	0.85 ± 0.00 ^c^	0.96 ± 0.01 ^b^	1.06 ± 0.00 ^a^
	POV (mmol/kg)	2.19 ± 0.10 ^f^	4.80 ± 0.00 ^e^	12.38 ± 0.20 ^d^	20.83 ± 0.04 ^c^	25.28 ± 0.81 ^b^	29.26 ± 0.85 ^a^
	p-AnV	11.49 ± 0.22 ^f^	13.42 ± 0.03 ^e^	21.98 ± 0.20 ^d^	42.37 ± 0.84 ^c^	64.69 ± 0.54 ^b^	122.24 ± 0.40 ^a^
	TV	20.26 ± 0.62 ^f^	32.61 ± 0.01 ^e^	71.50 ± 0.62 ^d^	125.70 ± 1.02 ^c^	165.81 ± 3.80 ^b^	239.29 ± 3.81 ^a^
180 °C	AV (mg/g)	0.82 ± 0.01 ^e^	0.82 ± 0.00 ^e^	0.85 ± 0.00 ^d^	0.89 ± 0.00 ^c^	0.99 ± 0.01 ^b^	1.09 ± 0.00 ^a^
	POV (mmol/kg)	2.19 ± 0.10 ^c^	2.60 ± 0.09 ^c^	8.57 ± 0.40 ^a^	7.58 ± 0.19 ^b^	8.26 ± 0.02 ^a^	7.33 ± 0.19 ^b^
	p-AnV	11.49 ± 0.22 ^f^	24.90 ± 0.04 ^e^	62.32 ± 0.74 ^d^	96.86 ± 0.16 ^c^	123.54 ± 0.64 ^b^	172.16 ± 0.74 ^a^
	TV	20.26 ± 0.62 ^f^	35.31±0.41 ^e^	96.59±0.85 ^d^	127.20±0.91 ^c^	156.56±0.71 ^b^	201.47±0.01 ^a^

AV, POV, p-AnV and TV are the abbreviations of acid value, peroxide value, anisidine value, total oxidation value, respectively. Different superscript letters indicate a significant difference at *p*-value < 0.05.

**Table 4:** Changes in AV, POV, p-AnV and TV of HOSFO&SSO oil blend during heating.

		0 h	0.5 h	1 h	1.5 h	2 h	3 h
150 °C	AV(mg/g)	0.78 ± 0.01 ^c^	0.78 ± 0.01 ^c^	0.78 ± 0.00 ^c^	0.86 ± 0.00 ^b^	0.85 ± 0.00 ^b^	1.06 ± 0.01 ^a^
	POV(mmol/kg)	2.72 ± 0.09 ^f^	5.61 ± 0.37 ^e^	12.09 ± 0.08 ^d^	19.86 ± 0.25 ^c^	25.02 ± 0.45 ^b^	30.20 ± 0.75 ^a^
	p-AnV	2.81 ± 0.20 ^f^	4.13 ± 0.13 ^e^	6.57 ± 0.54 ^d^	20.95 ± 0.56 ^c^	36.13 ± 0.48 ^b^	63.76 ± 0.35 ^a^
	TV	13.70 ± 0.58 ^f^	26.56 ± 1.36 ^e^	54.94 ± 0.85 ^d^	100.39 ± 0.44 ^c^	136.22 ± 2.27 ^b^	184.57 ± 2.67 ^a^
180 °C	AV (mg/g)	0.78 ± 0.01 ^d^	0.78 ± 0.00 ^d^	0.78 ± 0.01 ^d^	0.85 ± 0.00 ^c^	0.89 ± 0.01 ^b^	1.13 ± 0.00 ^a^
	POV (mmol/kg)	2.72 ± 0.09 ^d^	2.87 ± 0.47 ^d^	8.02 ± 0.00 ^c^	9.18 ± 0.34b ^b^	8.39 ± 0.58 ^bc^	10.94 ± 0.17 ^a^
	p-AnV	2.81 ± 0.20 ^f^	11.75 ± 0.61 ^e^	30.44 ± 0.83 ^d^	46.49 ± 0.54 ^c^	60.95 ± 0.68 ^b^	96.12 ± 0.31 ^a^
	TV	13.70 ± 0.58 ^f^	23.24 ± 2.50 ^e^	62.50 ± 0.84 ^d^	83.19 ± 0.84 ^c^	94.52 ± 1.65 ^b^	139.87 ± 0.37 ^a^

AV, POV, p-AnV and TV are the abbreviations of acid value, peroxide value, anisidine value, total oxidation value, respectively. Different superscript letters indicate a significant difference at *p*-value < 0.05.

**Table 5:** Changes in AV, POV, p-AnV and TV of HOPNO&SSO oil blend during heating.

		0 h	0.5 h	1 h	2 h	3 h
150 °C	AV (mg/g)	1.46 ± 0.02 ^c^	1.63 ± 0.02 ^b^	1.59 ± 0.02 ^b^	1.65 ± 0.02 ^b^	1.80 ± 0.05 ^a^
	POV (mmol/kg)	2.06 ± 0.09 ^e^	6.99 ± 0.08 ^d^	15.31 ± 0.93 ^c^	24.65 ± 0.01 ^b^	26.80 ± 0.15 ^a^
	p-AnV	6.64 ± 0.01 ^e^	8.28 ± 0.13 ^d^	19.45 ± 0.16 ^c^	48.39 ± 0.86 ^b^	68.30 ± 0.30 ^a^
	TV	14.88 ± 0.35 ^e^	36.26 ± 0.43 ^d^	80.69 ± 3.56 ^c^	146.98 ± 0.92 ^b^	175.51 ± 0.30 ^a^
180 °C	A (mg/g)	1.46 ± 0.02 ^c^	1.77 ± 0.05 ^b^	1.84 ± 0.06 ^b^	1.86 ± 0.07 ^b^	2.16 ± 0.06 ^a^
	POV (mmol/kg)	2.06 ± 0.09 ^c^	6.86 ± 0.08 ^ab^	6.79 ± 0.17 ^ab^	6.57 ± 0.09 ^b^	7.12 ± 0.08 ^a^
	p-AnV	6.64 ± 0.01 ^e^	24.20 ± 0.21 ^d^	69.36 ± 0.79 ^c^	76.12 ± 0.91 ^b^	89.66 ± 0.66 ^a^
	TV	14.88 ± 0.35 ^e^	51.66 ± 0.10 ^d^	96.53 ± 1.47 ^c^	102.41 ± 0.56 ^b^	118.13 ± 0.33 ^a^

AV, POV, p-AnV and TV are the abbreviations of acid value, peroxide value, anisidine value, total oxidation value, respectively. Different superscript letters indicate a significant difference at *p*-value < 0.05.

## Data Availability

Not applicable.

## Supplementary Materials

The following are available online, Table S1: Fatty acid compositions (%) of vegetable oils used in this work; Table S2: The contents of 14 HAAs determined in vegetable oils; Table S3: The MRM parameters for 14 HAAs and internal standard (4,7,8-TriMeIQx).

## Sample Availability

Samples are available from Wei Liu on valid request.
