# Supplementary material for: Degradation of β-Carbolines Harman and Norharman in Edible Oils during Heating
Source: Molecules. 2021 Nov 20;26(22):7018. doi: 10.3390/molecules26227018 (PMC8623535; doi:10.3390/molecules26227018)
Supplement: Supplementary file 1 [file molecules-26-07018-s001.zip › molecules-1441641-supplementary.pdf]

Supplementary Files

Degradation of β-Carbolines Harman and Norharman in Edible Oils during Heating

Wei Liu [1,]*, Zhaoyu Yang [1], Lili Shi [1], Ziyu Cui [1], Yun Li [2,]*

[1] College of Food Science and Technology, Henan University of Technology, Lianhua Street, Zhengzhou 450001, China; 201892085@stu.haut.edu.cn (Z.Y.); shililislla@163.com (L.S.); cuiziyu155@163.com (Z.C.)

[2] Key Laboratory of Agro-Products Safety & Quality of the Ministry of Agriculture, Institute of Quality Standards & Testing Technology for Agro-Products, Chinese Academy of Agricultural Sciences, No.12, Zhongguancun South Street, Beijing 100081, China

* Correspondence: liuwei307@hotmail.com (W.L.); liyun01@caas.cn (Y.L.);

Tel.: +86-371-67758022 (W.L.)

Table S1. Fatty acid compositions (%) of vegetable oils used in this work.

	SBO	SFO	HOSFO	HOPNO	SSO
C16:0	10.85	6.55	3.71	5.85	9.63
C18:0	4.25	3.38	2.52	2.51	6.28
C18:1	26.90	27.68	84.36	75.71	40.98
C18:2	52.07	60.89	6.75	6.90	41.15
C18:3	4.84	0.23	0.41	1.25	−
C20:1	-	-	-	2.25	−
C22:0	-	-	-	3.29	−
C24:0	-	-	-	2.24	−
**ΣSFA**	15.10	9.93	6.23	13.89	15.91
**ΣMUFA**	26.90	27.68	84.36	77.96	40.98
**ΣPUFA**	56.91	61.12	7.16	8.16	41.15

Note: soybean oil (SBO), sunflower seed oil (SFO), high-oleic acid sunflower seed oil (HOSFO), high-oleic acid peanut oil (HOPNO) and sesame seed oil (SSO) were used to prepare blend oils. SFA, MUFA and PUFA are the abbreviation of saturated fatty acids, mono-unsaturated fatty acids and poly-unsaturated fatty acids, respectively.

Table S2. The contents of 14 HAAs determined in vegetable oils (μg/kg) [a].

HAA (μg/kg)	Soybean oil	Sunflower seed oil	High-oleic acid sunflower seed oil	High-oleic acid peanut oil	Sesame seed oil
AαC	ND[b]	ND	ND	ND	ND
MeAαC	ND	ND	ND	ND	ND
Trp-P-1	ND	ND	ND	ND	ND
DMIP	ND	ND	ND	ND	ND
Glu-P-2	ND	ND	ND	ND	ND
MeIQ	ND	ND	ND	ND	ND
MeIQx	ND	ND	ND	ND	ND
IQ	ND	ND	ND	ND	ND
PhIP	ND	ND	ND	ND	ND
4,8-DiMeIQx	ND	ND	ND	ND	ND
7,8-DiMeIQx	ND	ND	ND	ND	ND
Harman	ND	ND	ND	ND	789±9.01
Norharman	ND	ND	ND	ND	890±7.95
Trp-P-2	ND	ND	ND	ND	ND

[a] Not detected (ND).

[b] Note: heterocyclic aromatic amines (HAAs) were detected by LC-MS.

Table S3. The MRM parameters for 14 HAAs and internal standard (4,7,8-TriMeIQx).

HAAs	Precursor ion[M + H]$^+$(m/z)	Diagnostic Productions (m/z)	Cone voltage (V)	Collision voltage (eV)	Dwell time (msecs)
AαC	184.0	167.2	108	32	30
		140.0	108	32	
MeAαC	198.2	154.1	104	40	30
		127.1	104	45	
Trp-P-1	212.0	168.0	80	30	30
		195.2	80	40	
DMIP	162.9	147.3	90	45	30
		105.0	90	45	
Glu-P-2	185.2	131.1	80	40	30
		78.2	80	40	
MeIQ	213.1	198.0	100	35	30
		144.0	100	60	
MeIQx	214.1	199.0	100	40	30
		131.0	100	55	
IQ	199.1	184.0	100	40	30
		157.0	100	50	
PhIP	225.3	210.2	120	45	30
		183.2	120	50	
4,8-DiMeIQx	228.1	211.8	100	45	30
		160.0	100	40	
7,8-DiMeIQx	228.0	131.3	100	55	30
		213.2	100	40	
4,7,8-DiMeIQx	242.0	227.1	120	40	30
		145.0	120	50	
Harman	183.0	115.0	120	50	30
		168.3	120	40	
Norharman	169.2	115.0	100	45	30
		142.0	100	40	
Trp-P-2	198.0	154.0	60	40	30
		128.0	60	40	

Note: heterocyclic aromatic amines (HAAs) were detected by LC-MS.
